# Treatment of Diseases by Oxygen Gas

**Published:** 1863-02

**Authors:** Edwin Holmes


Treatment of Disease by Oxygen Gas.—From a letter on this subject, published in the *Cincinnati Commercial*, we take the following:—

"Surgeon George G. Shumard, Medical Director of Danville District, suggested and instituted the experiment of administering oxygen gas by inhalation. Having in my possession a copy of his official report upon the subject, I take the liberty of transmitting the following extracts from it, which I hope will be sufficient to show who originated the important experiment referred to:—

"November 29, 1862.—I have frequently had occasion, in the course of my medical practice, to observe the apparent great want of oxygen in the blood drawn from patients laboring under different forms of autumnal disease. Impressed with the belief that this deficiency was not merely apparent but real, and that blood, thus unfavorably constituted, if not the cause of disease, could not otherwise than exercise a very prejudicial influence upon the system, I several years ago instituted a series of careful comparisons between healthy blood and that drawn from patients laboring under different forms of autumnal disease, and succeeded in fully satisfying myself that such a deficiency really did exist, and that there was an excess of carbonic acid in the blood of all the cases examined. It therefore occurred to me that if oxygen gas could, by any means, be artificially supplied to the circulation, it might afford a valuable remedy in the treatment of autumnal and various other forms of diseases. It also occurred to me that the best channel for administering the remedy would be that which nature has herself established for the reception of oxygen—the lungs. I therefore resolved to try the experiment as soon as a favorable opportunity presented itself.

"In 1857 I was called to see a case of severe congestive chill, in which the patient, a man about 30 years of age, was cold and nearly pulseless. Active stimulants and other remedies usually employed in such cases were freely resorted to. A small quantity of nitrous oxide gas was also prepared, and administered to the patient by inhalation. Shortly afterward the pulse increased in volume, and in about one hour from the time of the inhalation the extremities became warm, and the patient recovered from his chill. As other remedies were here employed besides the gas, and may have exercised as important influence in relieving the patient, I concluded to await the result of other experiments before publishing the case.

"Shortly after this my duties called me to another portion of the United States, and I had no further opportunities for repeating the experiment until the 22d of the present month.

"Last summer, while acting as Medical Director at Huntsville, Alabama, I repeatedly urged the employment of the gas in the treatment of disease. The different medical officers stationed at that post were favorably impressed with the idea that it might be made a useful remedy; but, from some cause or other, the gas was not administered. I also requested Dr. Newman, a highly accomplished private physician of Huntsville, to employ the remedy in such cases as he might deem favorable for its use. A number of physicians in Cincinnati were also urged, a year ago, to administer the gas in cases of disease.

"On the 22d instant, a case of typhoid fever, (Case No. 1,) of a hopeless character, was reported from Danville General Hospital No. 3. As the patient was apparently dying, and could not, therefore, be in any way injured by the experiment, I resolved to try the effects of the gas. Assistant Surgeon Devindorf was accordingly directed to administer the gas to him immediately, which he did in the presence of Assistant Surgeons Samlere, Aichele, and Simpson. The results were so striking in character as to impress every one present favorably with the remedy. I may here remark that two of the medical officers present, who were at first decidedly skeptical upon the subject, upon witnessing the result of the first experiment immediately changed their opinions and became enthusiastically in favor of the remedy.

"As soon as the favorable results of the gas began to exhibit themselves in case No. 1, Assistant Surgeons Samlere, Aichele, Devindorf, Simpson, and Avery were directed to visit the different hospitals in Danville, and, after having carefully examined the worst cases of disease in each, to select such for experiment as were considered entirely hopeless. They accordingly reported to me cases Nos. 2, 3, 4, 5, 6, and 7, to all of which the gas was immediately administered. The reports of all these cases are now before you, and from them you will be able to judge whether this remedy is or is not worthy of more extensive trial.

"Without attempting an analysis of these cases, I will merely remark that all the patients to whom the remedy was administered were pronounced hopeless by their attending physicians, and that their judgment in the matter was fully confirmed by that of the committee appointed to examine the cases before the gas was inhaled; that striking improvement was observed in every case after the gas was administered; that under its influence warmth slowly returned to the extremities, after the most powerful difusible stimulants that could be given had failed to produce that result; that the pulse increased its volume, and became much more natural to the touch; that the delirium which had, in several cases, existed for weeks previously, entirely subsided; that the involuntary discharges from the bowels in all but one case ceased; that several of the cases, after lying for many hours delirious, or insensible, became rational, and conversed with those around them; that the countenance assumed a much more natural expression; that the livid spots upon the chest and abdomen, of two of the cases, changed to a light rose color, and commenced disappearing; that the patients all expressed themselves as feeling much better; that the effects of the gas were not merely temporary but permanent; that in the four cases that have died, life was apparently prolonged many hours by the remedy; and that three out of the seven supposed fatal cases are still living, and may yet recover.

"I propose to continue the experiments, and shall hereafter not confine them alone to cases that are considered hopeless.

"Although it has thus far been tried in only eight cases, the results are sufficient to prove that we have in oxygen gas a remedy of surprising power, and one that bids fair to be of great service hereafter in the treatment of almost every variety of disease.

"The gas was administered to all the cases in the form of nitrous oxide, which was made in the usual manner from nitrate of ammonia, by Prof. Brikford, of Danville, and Assistant Surgeon Semlere, U. S. V. For want of better apparatus it was administered to the patients from beef bladders, which answered the purpose moderately well.

"Although the oxygen was employed in these cases in the form of nitrous oxide gas, I would not propose to use it so in all cases. In cholera and severe cases of congestive chill, I am persuaded that oxygen gas, in its pure form, or slightly diluted with atmospheric air, would be better; nor would I hesitate to give it in any form of disease in which the vital powers are depressed, since the cases recorded show that it relieves delirium and irritation instead of producing them."

"Such are the facts of the case, which can be certified to, if necessary, by every medical officer stationed at this post. Since the above report was written, the gas has been administered to a large number of patients here, and in every case with good effect.

I am, sir, very respectfully, your obedient servant,

EDWIN HOLMES.

